# eButterfly: Leveraging Massive Online Citizen Science for Butterfly Conservation

**DOI:** 10.3390/insects8020053

**Published:** 2017-05-18

**Authors:** Kathleen L. Prudic, Kent P. McFarland, Jeffrey C. Oliver, Rebecca A. Hutchinson, Elizabeth C. Long, Jeremy T. Kerr, Maxim Larrivée

**Affiliations:** 1 Entomology, University of Arizona, Tucson, AZ 85721, USA; 2 Vermont Center for Ecostudies, Norwich, VT 05055, USA; kmcfarland@vtecostudies.org; 3 Health Sciences Library, University of Arizona, Tucson, AZ 85721, USA; jcoliver@email.arizona.edu; 4 Electrical Engineering and Computer Science, Oregon State University, Corvallis, OR 97331, USA; rah@eecs.oregonstate.edu; 5 Fisheries and Wildlife, Oregon State University, Corvallis, OR 97331, USA; 6 Daniel Smiley Research Center, Mohonk Preserve, New Paltz, NY 12561, USA; elong@mohonkpreserve.org; 7 Biology, University of Ottawa, Ottawa, ON K1N 6N5, Canada; jeremy.kerr@uottawa.ca; 8 Insectarium, Montreal Space for Life, Montreal, QC H1X 2B2, Canada; 9 Institut de Research en Biologie Végétale, Montreal, QC H1X 2B2, Canada

**Keywords:** biodiversity, butterfly, Citizen Science, crowd-sourced, *Danaus plexippus*, data management, pollinator conservation, Lepidoptera, Papilionoidea, *Vanessa atalanta*

## Abstract

Data collection, storage, analysis, visualization, and dissemination are changing rapidly due to advances in new technologies driven by computer science and universal access to the internet. These technologies and web connections place human observers front and center in citizen science-driven research and are critical in generating new discoveries and innovation in such fields as astronomy, biodiversity, and meteorology. Research projects utilizing a citizen science approach address scientific problems at regional, continental, and even global scales otherwise impossible for a single lab or even a small collection of academic researchers. Here we describe eButterfly an integrative checklist-based butterfly monitoring and database web-platform that leverages the skills and knowledge of recreational butterfly enthusiasts to create a globally accessible unified database of butterfly observations across North America. Citizen scientists, conservationists, policy makers, and scientists are using eButterfly data to better understand the biological patterns of butterfly species diversity and how environmental conditions shape these patterns in space and time. eButterfly in collaboration with thousands of butterfly enthusiasts has created a near real-time butterfly data resource producing tens of thousands of observations per year open to all to share and explore.

## 1. Introduction

Identifying species is a challenging skill that can require specialized training and years of experience. Human observers still outperform computer algorithms in species identification because of the need to process impressions of shape, size, color, and behavior under variable conditions all while comparing this with another list of possible species based on time and location [1]. This difficult task of identification is central to many scientific fields and critical to conservation and preservation efforts; and humans, not current computer vision algorithms, do this task accurately and efficiently. Without accurate knowledge of which species occur in a given area at a particular time or season, developing strategies to manage biodiversity will remain a formidable challenge [2].

Most data on species-level occurrence are gathered by direct observation in the field. This necessitates innovative approaches for wide-scale data collection and analysis, such as massive online citizen science initiatives [3,4]. For example, the National Phenology Network [5] and Plant Watch Canada [6] focus on phenology of a subset of plants and animals in North America; iNaturalist [7] focuses on single observations of all taxa across the globe and is one of the Global Biodiversity Information Facility’s (GBIF) [8] contributors; eBird [9] focuses on bird checklists around the globe and has the most extensive database of avian distribution and abundance.

The checklist approach utilized in the field of ornithology since the early 1950s [10] is a powerful way to monitor a specific taxon. In its essence, a checklist in the field of biodiversity monitoring constitutes a list of species and their abundance recorded at a particular location. While checklists can take several forms and collect various pieces of information, they will generally collect spatiotemporal information about the observations reported, such as the date, time, and effort measured in duration, number of people participating, and distance travelled. As such, the data arising from checklists can be standardized in terms of effort and thus compared in space and time, dramatically increasing the scientific value of monitoring data when compared to single species “dot on the map” monitoring efforts. Butterflies are well suited to checklisting; they are well documented, and identification tools such as field guides are readily available for all regions of North America.

For more than 300 years, the public has contributed to the understanding of butterfly taxonomy, ecology, biogeography, evolution, and conservation [11]. In North America, The Lepidopterists’ Society was originally founded as an inclusive scientific society of both amateurs and professionals and still has almost equal membership between people who earn their living as lepidopterists and those who pursue their passion for butterflies out of private interest [12]. eButterfly (www.e-butterfly.org) builds on this tradition and provides a web-platform for citizen scientists to submit their checklists and photographs of butterfly observations across North America via the Internet. eButterfly engages a vast network of human observers (citizen scientists), now over 5000, to report butterfly checklists and photographic observations using standardized protocols analogous to eBird and compatible with iNaturalist and GBIF. eButterfly’s mission is to harness the power of butterfly enthusiasts in an effort to better understand butterfly distribution and abundance across broad spatiotemporal scales and identify factors that influence these patterns.

Butterflies are ideal insects for citizen science research [13]. There are nearly 10,000 species linked to biotic processes at multiple trophic levels. As larvae, butterflies are herbivores and are an important food source for other animals in the community. As adults, butterflies are pollinators and are easily trackable on the wing. Much is known about butterfly larval host plants and adult nectar plant affiliations across North America thanks to the combined efforts of professionals and amateurs. Butterflies are diurnal, behaviorally and morphologically conspicuous, and abundant. All of these characteristics mean butterflies are easily observed, counted, and are among the most studied of free-living insect species. Butterflies such as the monarch (*Danaus plexippus*) even engage in long-distance migrations and in doing so, demonstrate the biological integration of seemingly disparate ecosystems. Most importantly, butterflies are sensitive environmental indicators, signaling changes in environmental processes and ecosystem health [14,15,16].

## 2. eButterfly is an Online Citizen Science Community at a Continental Scale

eButterfly (www.e-butterfly.org), launched by Max Larrivee and Jeremy Kerr at the University of Ottawa in 2012, uses standardized web-based protocols. Initially, eButterfly was limited to observations from Canada but quickly expanded to the United States with the addition of Kent McFarland at the Vermont Center for Ecostudies and Katy Prudic and Jeff Oliver, both from the University of Arizona. eButterfly is currently available to citizen scientists in English and French, with plans underway for adding Spanish.

Butterflies are perhaps the most popular group of insects and are frequently observed and admired by almost everyone. Approximately 68.6 million people watch wildlife around their homes, with 24.1 million enjoying wildlife photography and 21.3 million indicating that they specifically watch insects [17]. Wildlife observation is rapidly growing, and becoming more affordable as higher performing digital photography technology is making the documentation of wildlife easier. However, the data butterfly enthusiasts collect are often stored on personal computers, and in file cabinets, notebooks, or specimen collections, thus being unavailable for science, conservation, and education. eButterfly provides a way for butterfly observational data to be organized, verified, and shared.

eButterfly serves multiple stakeholders (e.g., butterfly enthusiasts, conservation managers, policy makers, scientists, educators) as an open data source to gather, organize, verify, and disseminate checklists of butterflies across North America. These data provide information on species occurrence, migration timing, community structure, and relative abundance at a variety of spatial and temporal scales. Through the process of informal science education, eButterfly participants become more familiar with scientific approaches, data collection and analyses by using standardized data-gathering techniques, exploring data through the website’s data visualization tools, and interacting with scientists. eButterfly provides a permanent repository for checklist observations and a method for keeping track of each participant’s butterfly observations, photographs, and collection effort.

eButterfly data are proving vital for scientific use in broad scale ecological and conservation research. eButterfly data are available in real-time, creating new opportunities for rapid integration of butterfly data with other kinds of information such as climate, land cover, plant phenology, or even the timing of butterfly species’ seasonal activities, such as migration. For example, spatial variation in land cover and climatic conditions strongly structure butterfly species assemblages [18], but new butterfly observations have enabled detection of how warming temperatures are shifting butterfly species composition across broad areas of North America [19]. As a conservation tool, eButterfly is being used to monitor butterflies locally and regionally by providing new data on butterfly distribution, phenology, and relative abundance. eButterfly data can also contribute to improved understanding and models of species distribution, which helps prioritize and manage areas for conservation [20]. eButterfly’s broad spatial and temporal coverage complements more intensive monitoring programs, such as the Ontario Butterfly Atlas [21], allowing scientists to generate new hypotheses and direct future research efforts based on large amounts of data, beyond the scope of a single principal investigator or whole institute. Importantly, all eButterfly data contributes to scientific knowledge by joining networked data available through larger global biodiversity initiatives such as Bison [22], Canadensis [23], and GBIF [8].

**Figure 1:**
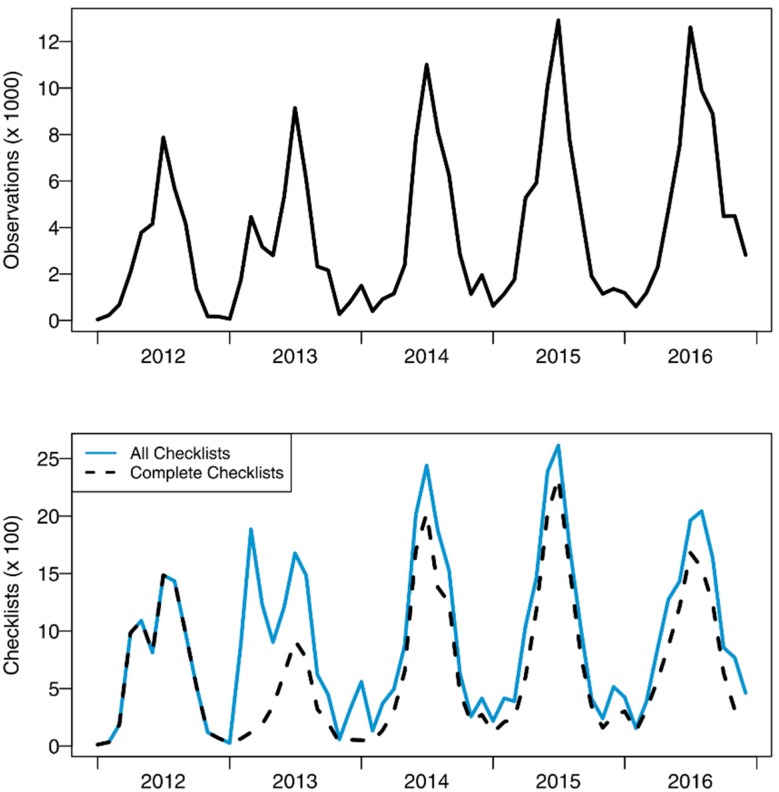
Monthly observations and checklists submitted to eButterfly since 2012. Over 39,000 checklists by 5500 registered participants have been gathered. Within those checklists there are 230,000 observations and 682 species. Note the heavy usage during summer followed by much lower reports in winter months due to the phenology of butterfly species in the northern hemisphere.

While it is continental in scope, the majority of eButterfly data still come from northeastern North America. Since its release in 2012, over 30,000 people have visited eButterfly. Over 5500 unique participants have entered data into eButterfly, from more than 28,000 locations across North America. The project has gathered over 230,000 observations for more than 39,000 checklists (Figure 1). eButterfly will also upload over 300,000 additional historical observations from museum collections in Canada and the United-States in 2017.

Funding to develop and operate eButterfly cyberinfrastructure was $60,000/year, making the cost per observation incurred by the science community very low. For example, eButterfly gathered over 60,000 observations in 2016, so each observation costs ~$1 (USA). With increasing citizen scientist contributions, eButterfly’s per-observation costs are likely to continue to decline. eButterfly relies on the work of its contributors, and measuring their contributions is essential; we estimate these efforts to have required (conservatively) 56,000 h. This in-kind donation of time and expertise by our citizen scientists is valued at ~$1,030,000 (USA) [24].

## 3. eButterfly’s Project Design Leverages Citizen Scientists’ Observations

eButterfly was created based upon two simple ideas. First, many people are passionate about observing butterflies and, second, every butterfly observation has potential research value for fundamental and conservation research. Even observations of common species from well-sampled areas have value for monitoring population change, phenology, and for other spatiotemporal studies. Using informal science education, eButterfly steers participants into standardized data collection and provides extensive resources to improve observers’ capacities for butterfly detection and identification. As with eBird [25] and other citizen science programs [26], eButterfly documents the presence or presumed absence of species as well as abundance through checklist data. To report butterfly observations, a web interface engages participants to submit observations through three interactive steps.

After logging into their eButterfly account to enter observations, participants are asked where they observed butterflies using an online mapping tool to select an existing or new location. Each citizen scientist’s existing location is stored in the database so that participants can more easily record new observations from established collection sites, which are also accessible using a drop-down menu or “favorites” list.

Step two asks participants to indicate which of four different protocols they used to count butterflies. Traveling and area counts are effort-based sampling protocols, which require submitting both the amount of time spent butterfly watching and the distance traveled or area searched. Timed counts lack the spatial component and only record the total amount of time spent observing butterflies. The fourth protocol is a less rigorous option, called ‘‘casual observation”, which requires only date, location, and species observed to describe the sampling event. Participants are encouraged to use the most rigorous protocols when possible.

The final step presents a checklist of the butterflies known from the state or province where the outing occurred. The participant fills in the number of individuals seen for each species and can upload photographs they captured of those species during the outing. Although “sight only” observations are accepted, eButterfly encourages participants to include photos with their observations for species verification. Observations are tagged as photograph, specimen, or sight observations. Other ancillary information such as counts of each life stage, host plants, sex, behaviors, and other notes, can also be added. At the end of the checklist, participants are presented with a final question that asks if they are submitting a checklist of all butterflies observed during the count. This is an important question that allows data consumers to determine if this is a complete presence/presumed absence checklist, which is more informative than presence-only data for many emerging machine learning and other analytical approaches in biogeography and conservation research [27].

## 4. Data Quality and Quantity Can Be Improved and Managed in Concert

Data collection design is critical to any successful scientific endeavor. Citizen science project designs vary in their intent and ability to collect high quality data. Many citizen science programs are focused on informal learning outcomes and participant experience rather than producing data that can be used for scientific inquiry. For eButterfly, project design is based on the formation of scientific questions, followed by the recruitment of participants to collect the data necessary to answer those questions. eButterfly focuses on scientific inquiry in changes in biodiversity, effects of climate change, and butterfly conservation from regional to continental scales [14,28,29]. Specifically, we are interested in how climate and land use change alters butterfly distributions, abundances, phenologies, and community structure in space and time. Our protocols and participant incentives encourage this data collection priority over others, such as new or rare species discovery. Open data and community vetting improves data quality. eButterfly is building a network of regional experts to verify each observation submitted. These experts are often “super-users” of eButterfly, with years of expertise on local butterfly species and their phonologies.

eButterfly encourages participants to submit photos of their observations as vouchers for species verification. Photographs are processed in a way that is comparable to how a natural history museum traditionally works with a specimen. The eButterfly participant is analogous to the collector, the regional data expert is analogous to the determiner, and eButterfly acts as the curator. Regional data experts validate or suggest corrections to observations with and without photographs based on species’ known ranges and phenology. Regional data experts can add a new determination for the observation base, which is held in the database as the correct identification. Our regional experts also vet submissions of collection specimens databased through the eButterfly interface, sometimes improving the data quality of the museum collection through our verification process. Once verification is made, the participant is alerted and has the opportunity to respond or correct their field identification. Additionally, eButterfly participants often report and comment on others’ observations, especially if they are incorrect, and this provides another mechanism for increased data quality.

To date, our vetters have reviewed over 190,000 butterfly observations. As eButterfly grows, we will build filters to automatically flag unusual records outside reported ranges and flight times to help streamline the data verification process as the data quantity exceeds the capacity for our regional experts. This combination of human computation and artificial learning algorithms has worked well in keeping data quality high in eBird as they expanded to millions of observations per year [30]. In the future, we hope to partner with experts in pattern recognition and machine learning to further automate butterfly species identifications from images [31].

Engaging citizen scientists is critical for increasing data quality and quantity. The data collection protocols and participation incentives of eButterfly engage large numbers of participants with a range of expertise from highly trained observers to young students, similar to other citizen science programs [1,25]. eButterfly particularly targets people with high levels of butterfly identification expertise, who in their spare time, collect, photograph, and watch butterflies for recreation. Many citizen science projects struggle to engage participants, and it can be especially hard to sustain participation when projects incorporate little or no rewards. In these instances, the number of participants generally plateaus once the threshold of like-minded individuals is reached [4,25]. eButterfly uses a variety of participant incentives such as life lists, year lists, species distribution maps and phenology graphs, and other tools to continue building and maintaining participation. We also try to communicate frequently with our super-users, who contribute the majority of data, and feature them in news stories and social media.

While crowd-sourced programs like eButterfly collect large amounts of data with broad spatiotemporal coverage, the data are influenced by more noise and bias than data collected through rigorous protocols conducted exclusively by expert-level observers. Imperfect detection of organisms is a pervasive source of noise in biodiversity surveys [32,33], affecting both citizen science and more specifically targeted data collection. In citizen science data, the effects of imperfect detection may be more variable due to differences in observers’ skill and expertise, but extensions of common methods to correct for detectability can account for this variability [34]. Citizen science data are more affected by spatiotemporal sampling bias than studies with careful sampling designs [35], and analyses of these data benefit from modeling methods that account for this bias [36,37].

**Figure 2:**
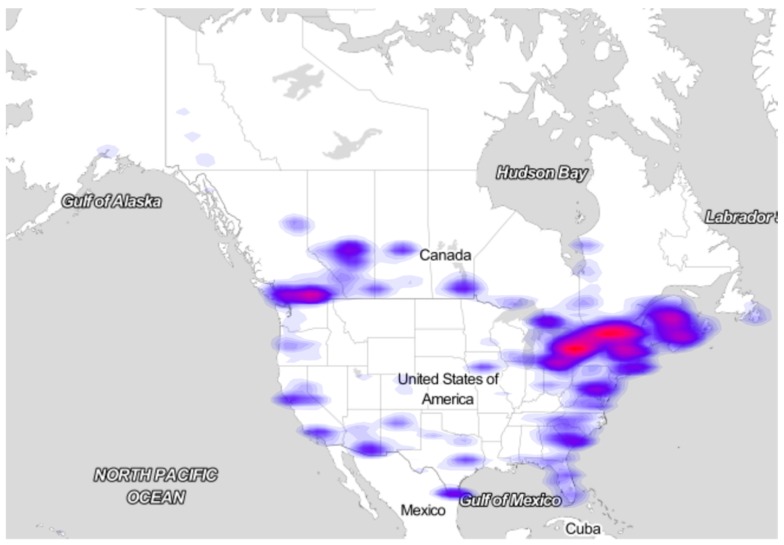
Spatial density map of all checklists reported to eButterfly. High density of checklists is shown in red, with areas of low density shown in blue. Note the spatial bias in relation to areas of high human population density such as urban centers and corridors.

Sampling effort by participants is an important source of bias in eButterfly data. eButterfly data are more robust near urban centers in the Northeast than in areas with sparse spatial coverage, such as in the Midwest (Figure 2). Similar to other crowd-sourced biodiversity projects (e.g., iNaturalist and eBird), observations tend to be clustered around major urban areas. Like eBird [4], we attempt to capitalize on butterfly watchers’ strong desire to find and identify as many species as possible by providing tools that gamify butterfly watching. For example, we provide a system that automatically displays participants’ life lists at multiple spatiotemporal scales and a ‘Top 100’ list that displays each citizen scientist’s scores for the total number of species, photographs and checklists. Additionally, we have started collaborating with Adventure Scientists [38] to specifically target remote areas that are unreachable by most citizen scientists. Adventure Scientists recruits and trains backcountry specialists to collect data for a variety of scientific ventures. We have also targeted specific under-collected and under-engaged regions, such as the Arctic, in 2011 and 2012 (Larrivée and Kerr, unpublished data). Finally, we control for several aspects of this data collection bias by including appropriate covariates in analytic models. Variation in detection rates is modeled as a function of observation effort, both the total time and the distance or area of the count, underscoring the importance of steering citizen scientists to these more rigorous protocols.

eButterfly has found a balance between data quantity and quality that has allowed us to gather a volume of useful butterfly data at spatial and temporal scales previously unattainable. Our experience has shown us that a simplified protocol and incentives for volunteer participation are critical for the recruitment and retention of large numbers of volunteers. We found crowd-sourcing verification is critical to both our data quality and participant engagement. Previous methods of data analysis may not always be suitable for use even after verification and vetting, so new analytical approaches are being created in cooperation with other ecologists, statisticians, and computer scientists.

## 5. eButterfly Provides Presence/Presumed Absence Data for Butterfly Conservation

eButterfly is a source of presence/presumed absence data, not just presence-only data. Presence-only data, which includes much of the data from natural history specimen collections, can be less informative in complex modeling such as species distribution models (SDMs) [27,39]. These models play a critical role in conservation decision-making and ecological or biogeographical inference, but can lead to suboptimal conservation outcomes and misguided theory if the underlying data do not suit the intended application. Building models with unsuitable data can waste valuable resources and deliver outputs that do not inform conservation and management decisions. eButterfly data collection protocols offer more and better modeling outcomes for conservation and ecology.

**Figure 3:**
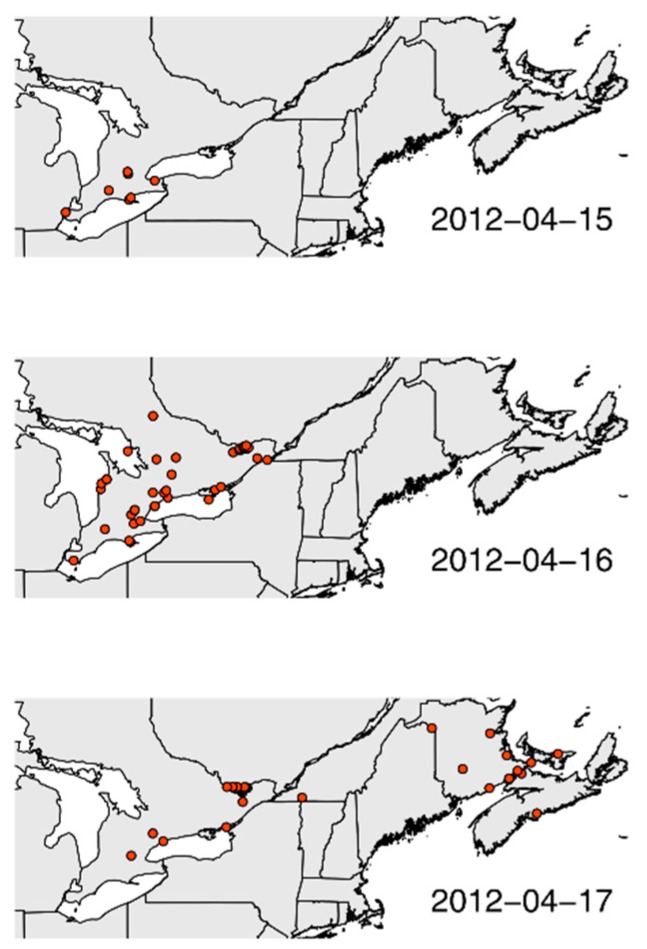
Observations of the red admiral (*Vanessa atalanta*) reported to eButterfly over three days in April 2012 in eastern Canada.

With these data, we are able to address previously difficult ecological and conservation questions. A long-standing challenge in insect conservation has been the difficulty of studying annual cycles for species that undergo broad-scale and complex geographic movements. Massive online citizen science web-platforms have started to make these movements trackable. The migration of the red admiral (*Vanessa atalanta* Nymphalidae) in Spring 2012 provides an example. Benefiting from an historically warm winter and spring in their southern United States overwintering grounds [40,41], record numbers of red admiral butterflies migrated northward following warm southern wind currents in early spring. Observations of red admirals were submitted to eButterfly so rapidly and over such a broad area that we could track the leading edge of the migration in real-time across southern Ontario, Canada. Using observations captured by participants from 15–17 April 2012, we estimated that the migrant population approached ~300 million individuals migrating over an 800,000 km^2^ area (Figure 3). The estimated migration speed of red admirals closely matched wind speeds recorded in the region (mean = 26 km/h ± 7 km/h) (Larrivée et al., unpublished data).

**Figure 4:**
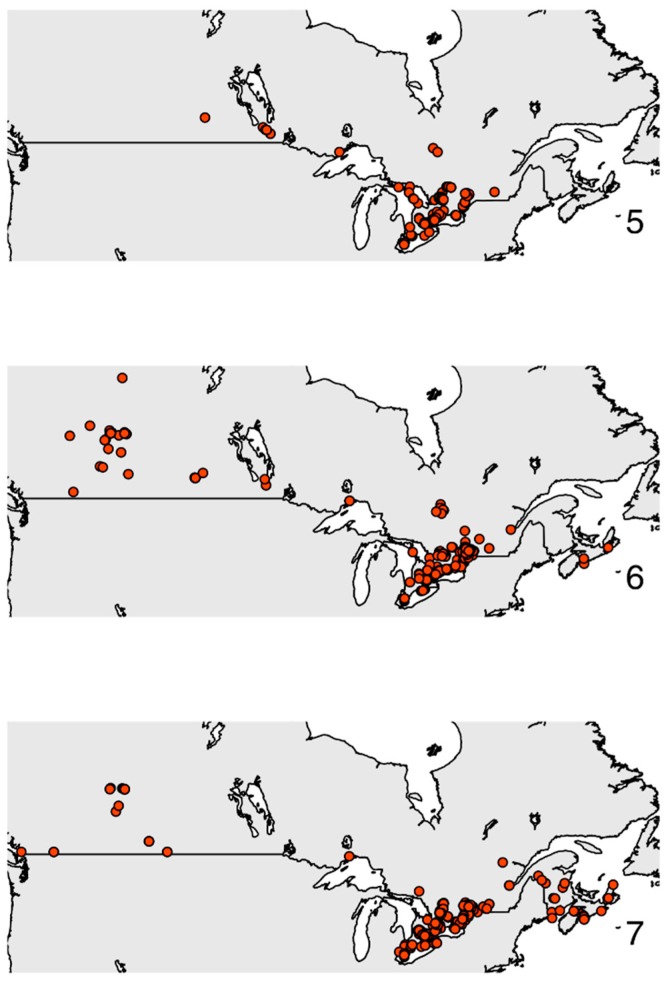
Observations of the monarch (*Danaus plexippus*) reported to eButterfly across Canada over three months in 2012. Note the appearance of monarchs in western Canada in June and July.

Another example of a large spatial movement captured in near real-time was recorded with the monarch butterfly (*Danaus plexippus* Nymphalidae) across Canada. This species appeared unexpectedly and in high numbers during the summer of 2012 in Alberta, Canada (Figure 4). Monarch butterflies normally range across the eastern half of Canada, but beginning in early summer, participants began reporting large numbers of monarchs farther west, in the province of Alberta, documenting what may be a rare east to west dispersal. These rare dispersal events may be a mechanism for gene flow between eastern and western monarch populations, which show little genetic subdivision across North America [42]. However, since eButterfly was only collecting data in Canada in 2012, we cannot rule out a south to north dispersal coming from the United States to Alberta. This uncertainty illustrates the importance of a continental approach to understanding species dispersal and movement in space and time. The Great Basin, in the western United States, is undersampled in eButterfly and other monarch citizen science programs such as Journey North [43], so the movement of monarchs in the summer of 2012 remains shrouded in uncertainty. Despite an abundant monarch population in the northern latitudes (southern Canada) over the summer of 2012, milkweed (*Asclepias* spp.) abundance is low or absent where monarchs were recorded in 2012, and fewer monarchs were detected returning to southern latitudes (central Mexico) relative to historical expectations [44].

Environmental change has potential to shift phenologies and impact butterfly survival and reproduction, so establishing multi-species migration and distribution monitoring capability is increasingly important and complements remote sensing-based measurements for ecology [45,46]. Mass migrations and range expansions of butterflies often involve important pollinator or pest species. Real-time measurement of the timing and magnitude of migration and range expansion, such as those provided through eButterfly, allows scientists to study the impacts of global change on butterflies and relate these findings to conservation priorities.

## 6. New Collaborations and Opportunities are on the Horizon

Despite the growing volume of species occurrence data collected by eButterfly, species observations are often sparsely distributed in space and through time, similar to museum collection data. This has motivated us to begin developing models of species occurrence by relating environmental features that may help elucidate a species’ preferred niche (e.g., habitat, climate, elevation) to observational data. These models can then be used to make statistical predictions of occurrence at unsampled locations and times. To facilitate this process, eButterfly observations have been linked through observation location and time to a large number of local environmental variables, such as remotely sensed habitat information from the National Land Cover Database, and vegetation phenology from NASA’s Moderate Resolution Imaging Spectroradiometer. We use these data to estimate species distributions designed to utilize both the broad extent and fine resolution information in the eButterfly database. Moving forward, we are exploring ways to estimate year-round distributions for all North American butterfly species with a wide variety of distinct migration pathways and local habitat associations. With this information, scientists and land managers will be better able to identify, prioritize, and coordinate conservation actions across broad landscapes.

Butterfly conservation is becoming more important as the general public becomes more interested in their success and survival, and as butterflies are increasingly a model system for understanding threats to insect pollinators. A significant strength of eButterfly as a conservation tool lies in its near real-time collection and dissemination of data, the reliability of which is often traceable to associated photographic vouchers. We are working on how to leverage the increasing quantity of photographic data on eButterfly to improve data verification, collection, and modeling. Up-to-the-minute butterfly observations could be coupled with other disparate data sources to provide powerful on-the-ground conservation tools. For example, a visualization tool which alerts interested stakeholders which species could be vulnerable to pesticide usage could help growers and landowners to weigh the risks of particular management options. Other conservation tools may alert stakeholders about nectar and host plant requirements for local butterfly species assemblages at different times of year, providing on-demand updates for land management decisions.

The eButterfly database structure and code has been instrumental in the development of other entomological citizen science monitoring programs, namely Bumblebee Watch [47] and Mission Monarch [48]. eButterfly has a similar data collection protocol to eBird, so efforts are underway to leverage the data of both initiatives to produce conservation data products with both birds and butterflies in mind. eButterfly has been actively collaborating with Nature’s Notebook sponsored by the National Phenology Network with the Nectar Connector campaign [49] to monitor the phenology of nectar plants across the United States and to relate plant phenology with butterfly phenology at various locations.

## 7. Conclusions

The power of eButterfly and other massive online citizen science programs lies in the strength and diversity of its participants and stakeholders. Anyone with an interest in butterflies can participate—from the new enthusiast, to the backyard gardener, to the seasoned expert. As more participants submit data, an environment of sharing and free data exchange will become the norm between butterfly enthusiasts, scientists, and conservationists. There is an urgent need for butterfly enthusiasts to begin to explore new and uncharted territory. Visiting under-sampled habitats will improve understanding of species-habitat relationships and help prioritize management interventions.

Global climate change, land conversion, and human population growth are presenting significant conservation challenges that require understanding processes at continental and global scales. Monitoring systems across full annual cycles in near real-time to identify current and impending threats and to evaluate the outcomes of conservation actions can only be done with massive online, crowd-sourced efforts at multiple spatial and temporal scales. For terrestrial insect pollinators and herbivores, eButterfly is well poised to provide this information.

The ability to create, manage, and manipulate vast, real-time data resources is changing how biological research is conducted and has the potential to revolutionize conservation practices. Critical to these new tools is the cyberinfrastructure of eButterfly, which depends on database, networking, and computational technologies. eButterfly is an excellent example of how these technologies can be used to grow, manage, and sustain a community of citizen scientists, whose cooperation may be the only way to gather large amounts of data needed to detect key biological impacts of broad-scale environmental change. eButterfly synthesizes massive volumes of information in real-time, providing the critical data that is necessary to answer questions about pollinator diversity and distributions and to address potential issues on how anthropogenic and environmental factors affect said diversity. With a continental perspective on insect dynamics, we can better engage policymakers and stakeholders and propose reasonable, understandable, and accountable management solutions. We believe the result of such efforts will be a better understanding of the critical conservation issues facing insect communities across the planet.

## Supplementary Materials

Figure 1, Figure 2, Figure 3 and Figure 4 were produced in R [50], along with the ggplot [51] and ggmap [52] packages. R code used to generate the figures is available at https://github.com/jcoliver/ebutterfly-graphing.
